# Integrative Taxonomy Clarifies Species Limits in Two Closely Related Solitary Wasps: *Pachymenes ater* and *Pachymenes ghilianii* (Hymenoptera: Vespidae: Eumeninae)

**DOI:** 10.3390/insects17010078

**Published:** 2026-01-09

**Authors:** Wellington Ferreira, Rodolpho Menezes, Matheus Viana, Marcel Hermes

**Affiliations:** 1 Centro de Estudos em Sistemática e Biologia de Insetos, Centro de Biodiversidade e Patrimônio Genético, Instituto de Ciências Naturais, Universidade Federal de Lavras, Lavras 37203-202, Brazil; marcelhermes@ufla.br; 2 Instituto Federal de Educação, Ciência e Tecnologia Baiano—Campus Guanambi, Zona Rural, Distrito de Ceraíma, Guanambi 46430-000, Brazil; 3 Departamento de Ciências Biológicas, Universidade Estadual de Santa Cruz, Ilhéus 45662-900, Brazil; rstmenezes@gmail.com (R.M.); matheusmakio120@gmail.com (M.V.)

**Keywords:** species delimitation, molecular phylogenetics, Neotropical wasps, morphology, mitochondrial markers

## Abstract

Species delimitation in morphologically conserved groups poses a significant challenge for traditional taxonomy. In the Neotropical wasp genus *Pachymenes* (Hymenoptera: Vespidae: Eumeninae), two putative sister species—*Pachymenes ater* and *P. ghilianii*—have historically been distinguished by a limited set of ambiguous morphological characters, often complicated by intraspecific variation and overlapping geographic distributions. Here, we apply an integrative taxonomic framework combining morphological, molecular, and geographic evidence to test species limits between these two taxa. We reassess previously proposed diagnostic traits and explore the utility of additional characters across multiple lines of evidence. Our results indicate that some morphological characters traditionally used for identification are insufficient for clear separation of species, while other, previously overlooked traits offer improved resolution. Molecular data further support species distinction and provide new insights into the evolutionary relationship between *P. ater* and *P. ghilianii*. This study highlights the importance of combining diverse data sources for robust species delimitation in solitary wasps and contributes to a better understanding of species boundaries within Eumeninae.

## Simple Summary

Telling closely related species apart can be difficult when they look very similar on the outside. This problem occurs in a group of solitary wasps found in Central and South America, where two suspected sister species have long been hard to separate because they share many body features and often live in the same regions. In this study, we investigated whether these two wasps truly represent separate species by examining them in several ways. We looked closely at body structures, including some features that had not been considered before, and compared them with modern genetic information and data on where each form is found. We discovered that some of the traits traditionally used to identify the species are not reliable, but other, previously overlooked features help distinguish them more clearly. The genetic evidence also supports the idea that they are two separate species and reveals how they are related to each other. By bringing together different types of information, our study shows a more accurate way to define species in groups that appear very uniform. This approach can improve the classification of solitary wasps and help guide future research on their diversity and conservation.

## 1. Introduction

Integrative taxonomy has emerged as a powerful framework for species delimitation, advocating the use of multiple and complementary data sources to achieve more accurate taxonomic decisions [1,2,3,4]. Under this approach, biodiversity is investigated through a multifaceted perspective that incorporates biological data from morphology, molecular biology, behavior, and ecology to address questions concerning the nature and boundaries of species [1].

Despite methodological and logistical challenges, integrative taxonomy has gained substantial traction in recent years. Within Hexapoda, studies focused on Hymenoptera represent a significant portion of integrative taxonomic literature [5,6,7,8,9,10]. Some studies are of particular interest [7,9,11,12,13], which apply integrative methods to Vespidae. Nonetheless, many lineages within this family remain poorly studied from this perspective, including the subfamily Eumeninae.

The genus *Pachymenes* de Saussure is a Neotropical group of solitary wasps [14,15], reported in Brazil from Atlantic Forest fragments, highland regions, and the Cerrado [16,17]. A comprehensive taxonomic revision of *Pachymenes* was conducted in recent years [15], recognizing 18 valid species and presenting a phylogenetic hypothesis. In their treatment, the authors discouraged the recognition of subspecies, aligning with earlier critiques of infraspecific taxa based solely on cuticular coloration in vespids [18,19].

Although their study clarified species relationships within the genus, certain taxonomic issues remain unresolved. One such case involves the delimitation of *Pachymenes ater* de Saussure and *Pachymenes ghilianii* (Spinola), recovered as sister species in their phylogenetic analysis and united by a single homoplastic trait—punctation on the second metasomal tergum [15]. Identification of these species using the available key can be problematic due to overlapping or ambiguous morphological characters. For instance, the most accessible character applicable to both sexes—development of the pronotal carina—is described as weak or medially restricted in *P. ater* and more strongly developed across the dorsal pronotal surface in *P. ghilianii*. However, the subjective nature of this distinction and the absence of illustrations render it difficult to apply reliably, especially without comparative material at hand.

A second character, coarse punctation on the apical half of the first metasomal tergum (in *P. ater*) versus smooth or finely punctate (in *P. ghilianii*), also lacks diagnostic clarity, as both conditions are described as “usually” present, with no mention of potential overlap. Furthermore, variation in cuticular coloration adds to the confusion. While *P. ghilianii* typically displays a yellowish-brown integument with black markings, melanic forms closely resemble *P. ater*, which is generally uniform in dark coloration with minimal markings [14,15].

The potential for sympatric occurrence adds a further layer of complexity. Distributional records suggest overlap between these species, and in some localities, specimens of both are morphologically similar with respect to color and putatively diagnostic traits [14,15]. As a result, species boundaries between *P. ater* and *P. ghilianii* remain unclear.

To date, few studies have explored such frameworks in solitary vespids. Taxonomic work in Eumeninae remains reliant on discrete morphological characters, leaving species hypotheses under-tested. A reassessment of current morphological criteria, alongside the inclusion of additional data (e.g., molecular markers, ecological preferences), is necessary to improve species delimitation. Following the principles of integrative taxonomy [20,21], this study applies a multi-evidence approach to investigate the validity and limits of *P. ater* and *P. ghilianii*.

## 2. Materials and Methods

### 2.1. Sampling

Specimens from multiple localities were used in order to cover the geographic distribution of *P. ater* and *P. ghilianii*. Both dry-mounted and ethanol-preserved specimens (90%) were employed for molecular analyses (Table S1 in Supplementary Material—henceforth SM). Morphological studies were conducted exclusively on dry-mounted material (see SM). For phylogenetic analyses (see below), *Pachymenes sericeus* (de Saussure) was used as the outgroup.

Specimens were identified to species level following the key provided by Grandinete et al. [15], and comparisons were made with material previously identified by the same authors. The specimens were sourced from the Coleção Entomológica da Universidade Federal de Lavras (CEUFLA), and on loan from the following institutions: AMNH, American Museum of Natural History (New York, NY, USA—Dr. James Carpenter); DZSJRP-Hymenoptera, Coleção de Hymenoptera de São José do Rio Preto, Instituto de Biociências, Letras e Ciências Exatas, Universidade Estadual Paulista “Júlio de Mesquita Filho” (São José do Rio Preto, Brazil—Dr. Fernando Noll); RPSP, Coleção Entomológica Prof. J.M.F. Camargo, Faculdade de Filosofia, Ciências e Letras de Ribeirão Preto, Universidade de São Paulo (Ribeirão Preto, Brazil—Dr. Eduardo Andrade Botelho de Almeida); CESC/UNISC, Coleção Entomológica da Universidade de Santa Cruz do Sul (Santa Cruz do Sul, Brazil—Dr. Andreas Köhler) (see Supplementary Materials).

### 2.2. DNA Extraction, Amplification, Sanger Sequencing, and Sequence Edition

Thoracic muscle was extracted from both dry-mounted and ethanol-preserved specimens, following the removal of the head and forelegs. Specimens were then remounted, preserving the exoskeleton. DNA was extracted from the isolated thoracic muscle using the QIAquick^®^ Gel Extraction Kit (QIAGEN, Hilden, Germany). Two mitochondrial protein-coding genes were tested for amplification efficiency: Cytochrome c oxidase subunit I (COI), primers: CI-J-1718 [22] and CI-N-2191 [23]; and Cytochrome c oxidase subunit II (COII), primers: E2 [24] and COII1-2 [25]. Genbank accession numbers for specimens whose COI was amplified range from PX649439 to PX649454, and for COII from PX685873 to PX685884 (Table S3). Specific PCR conditions followed [26,27] (see also Table S2). PCR products were purified using the QIAquick^®^ PCR Purification Kit (QIAGEN) following the standard protocol and sent for sequencing to the Centro de Recursos Biológicos e Biologia Genômica (CREBIO), Universidade Estadual Paulista “Júlio de Mesquita Filho” (Jaboticabal, Brazil).

Sequence quality was evaluated using Geneious v7.1.3 [28]. Forward and reverse reads were assembled into contigs within the same software. Contigs were edited to remove sequencing noise and to identify ambiguous bases. All samples were processed, but only 17 yielded successful amplification for at least one marker (Table S3).

Independent alignments for each locus were performed using MUSCLE (Multiple Sequence Alignment) with default parameters [29]. Alignments were visualized and edited in Mesquite v3.31 [30], which was also used to generate the concatenated dataset.

Partitioning and molecular evolution model selection were performed using PartitionFinder2 [31], applying the Bayesian Information Criterion (BIC). The molecular evolution models for each gene are listed in Table S4. Phylogenetic analyses were conducted separately for each locus and also using the concatenated dataset (see below).

### 2.3. Phylogenetic Analyses

Phylogenetic reconstructions were conducted using maximum parsimony (MP), maximum likelihood (ML), and Bayesian Inference (BI). MP analyses used equal character weighting [32]. Heuristic searches were performed in TNT v1.1 [33] using “New Technology Search” with the following settings: Sectorial Search [34] in default mode, Ratchet [35] with 200 iterations and perturbation phase set to 8 (for both up- and down-weighting), Drift [34] with 20 cycles, and Tree Fusing [34] with 10 rounds. The procedure was repeated until the shortest tree length was recovered at least 100 times. Random seed was set to 0. Gaps were treated as missing data. Clade support was assessed by bootstrap with 1000 replicates under traditional search.

ML trees were inferred using Garli 2.01 [36] under default settings. Branch support was assessed via 1000 bootstrap replicates and summarized as a 50% majority-rule consensus tree in PAUP* v4 [37]. BI analyses were performed in MrBayes v3.2.3 [38], using two independent runs of four chains for 50 million generations, sampling every 1000 generations, with 20% burn-in. Burn-in, convergence, and stationarity were assessed using Tracer v1.6 [39]. Trees resulting from MP analyses were visualized and edited in WinClada v1.00.08 [40], while ML and BI trees were processed in FigTree v1.4.0 [41].

### 2.4. Morphological Character Reassessment

Morphological character reassessment was based on both the phylogenetic results and direct comparative analysis among studied specimens. Comparative analyses were conducted between specimens from the same locality and/or corresponding to the same molecular clade, as well as between specimens of different clades. Additionally, diagnostic characters for the genus *Pachymenes* and both studied species, as previously proposed in the literature [15,42], were reevaluated to assess their variation and taxonomic reliability.

Special attention was given to characters traditionally used in keys and species diagnoses, including those related to the coloration of the metasoma and appendages, body punctation and sculpture, male genitalia, clypeal and cephalic morphology, mesosomal configuration, and wing venation. Morphological variation was interpreted in light of the molecular results, especially to evaluate whether the observed variation corresponded to intraspecific polymorphism or indicated interspecific divergence.

For diagnostic purposes, emphasis was placed on characters with minimal ontogenetic variation and low environmental plasticity. Whenever possible, we prioritized the use of discrete, qualitative characters with high interspecific stability, minimizing the reliance on continuous or highly variable traits.

Terminology for general external morphology and for male genitalia followed traditional works on Eumeninae [43,44]. Some terms referring to the mesepisternal region were adapted [45]. The abbreviations F1–F11 were used for antennal flagellomeres, while T1–T7 and S1–S7 refer to metasomal terga and sterna, respectively.

Morphological traits were reanalyzed in light of the phylogenetic clades recovered in both separate and combined analyses, as well as the results of the species delimitation tests. For each clade, we verified the consistency of diagnostic characters that may support species recognition.

### 2.5. Environmental Space, PCA, and Niche Overlap

We used occurrence records of both species (Table S1). From the monthly TerraClimate dataset (1958–2024; https://www.climatologylab.org/terraclimate.html, accessed on 15 September 2025), including minimum temperature, maximum temperature, and precipitation, we derived the 19 standard bioclimatic variables (BIO01–BIO19) using the biovars function in the dismo package [46]. To reduce multicollinearity, we applied Pearson correlation analysis and excluded variables with |r| ≥ 0.70 [47]. The remaining predictors were BIO01 (annual mean temperature), BIO02 (mean diurnal range), BIO03 (isothermality), BIO12 (annual precipitation), and BIO15 (precipitation seasonality) (Table S2). This five-variable subset jointly captures thermal averages, intra-daily and seasonal temperature variation, and the hydrological regime, while minimizing redundancy among predictors.

The accessible area (M) for each species was defined as the minimum convex polygon (MCP) encompassing all occurrences and buffered by 100 km. Random background points were sampled within each species-specific M (M_1_, M_2_) and within their union (M_1_ ∪ M_2_). The union area was used to calibrate the environmental space, while the individual M represented species-specific environmental availability. Environmental space was summarized using a principal component analysis (PCA) based on the background of M_1_ ∪ M_2_ (centered and scaled data), and both occurrence and background points were projected onto the first two principal components (PC1–PC2).

In the PCA-environment framework, we computed kernel density grids (R = 100) [48], as implemented in the ecospat package [49], resulting in normalized environmental utilization surfaces. Niche overlap was quantified using Schoener’s D, and overlap levels were classified according to Engler’s framework: 0–0.2 (no overlap), 0.2–0.4 (low), 0.4–0.6 (moderate), 0.6–0.8 (high), and 0.8–1.0 (very high) [50]. Niche equivalence was tested using permutations within the combined background (H_0_: niches are indistinguishable in the PCA-env), while directional similarity was assessed in both directions (A → B and B → A) [51]. For each test, the target species’ niche was kept fixed and the source species’ occurrences were randomized within M_B, with 1000 permutations.

Finally, to evaluate the robustness of our results, we repeated the entire workflow using only BIO01 and BIO12 (annual mean temperature and annual precipitation, respectively). We then compared Schoener’s D, test *p*-values, and the cumulative variance explained by PC1 + PC2 between both analyses.

## 3. Results

### 3.1. Molecular Data

Sequences were obtained for 16 samples (COI; 472 bp) and 12 samples (COII; 617 bp). These were independently aligned and then concatenated into a final matrix of 17 terminals and 1089 characters. Among the 1089 characters, 841 were constant, 185 were parsimony-informative, and 63 were variable but uninformative.

### 3.2. Phylogenetic Analyses

**Figure 1:**
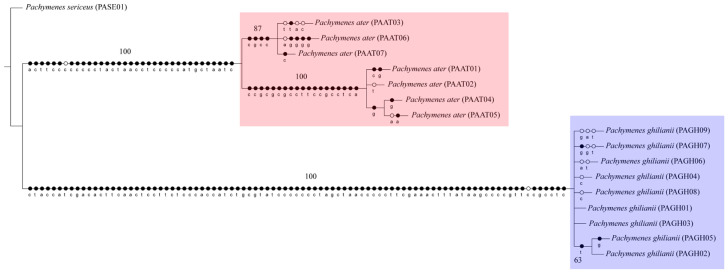
Strict consensus of five equally parsimonious cladograms (length = 306; consistency index = 0.90; retention index = 0.96) based on equal character weighting of the concatenated dataset (COI + COII). Support values were estimated using bootstrap (1000 replicates). Black circles represent synapomorphies; white circles represent homoplasies.

**Figure 2:**
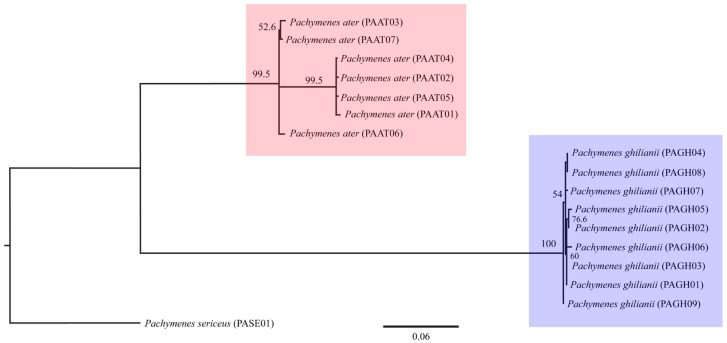
Maximum likelihood phylogeny based on 1089 bp of mitochondrial genes COI and COII. Numbers on branches indicate bootstrap support.

**Figure 3:**
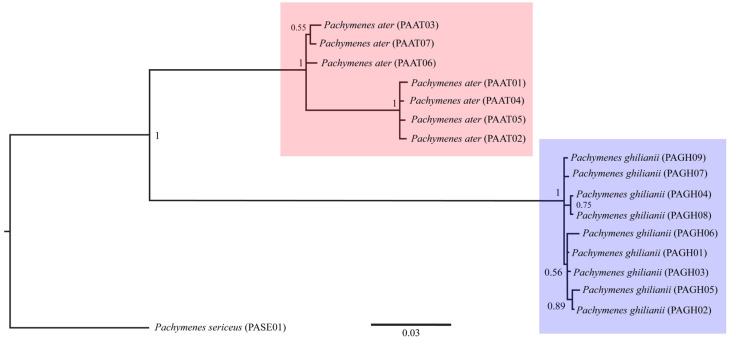
Bayesian inference phylogeny based on 1089 bp of mitochondrial genes COI and COII. Numbers on branches indicate posterior probability values.

The three phylogenetic methods (MP, ML, and BI), both the concatenated (COI + COII) and single-locus datasets consistently recovered *P. ater* and *P. ghilianii* as reciprocally monophyletic with strong statistical support (Figure 1, Figure 2 and Figure 3 and Figures S1–S6). Maximum parsimony yielded five equally parsimonious trees that, despite limited internal resolution, delineated two well-supported species-specific clades and revealed two subgroups within *P. ater*. Maximum likelihood similarly supported monophyly in all datasets, though it recovered different internal topologies and consistently identified a highly divergent lineage within *P. ater* (PAAT01, PAAT02, PAAT04, PAAT05). Finally, Bayesian inference also strongly supported monophyly for both species in all analyses, with internal relationships largely congruent with MP and ML for *P. ater*, but more variable for *P. ghilianii*. Together, these results robustly confirm species-level distinctiveness while revealing considerable genetic structuring within *P. ater*.

### 3.3. Reassessment of Morphological Characters

**Figure 4:**
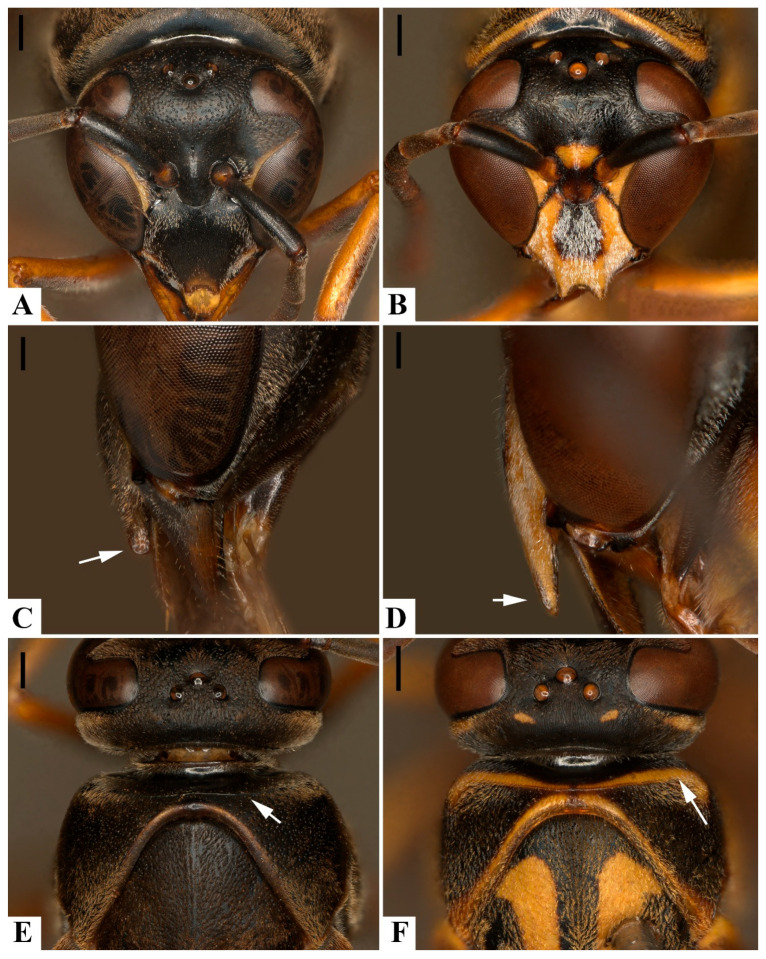
Diagnostic structures for the identification of *Pachymenes ater* de Saussure and *Pachymenes ghilianii* (Spinola): (**A**,**B**) Head, frontal view. (**C**,**D**) Clypeus, lateral margin. (**E**,**F**) Pronotum, dorsal view. (**A**,**C**,**E**): *P. ater*; (**B**,**D**,**F**): *P. ghilianii*. Scale bars: 0.5 mm (**A**,**B**,**E**,**F**); 0.2 mm (**C**,**D**).

Males of the two species can be reliably distinguished by the shape of the clypeus and genitalia (Table 1). The male clypeus provides the most accessible diagnostic feature: in *P. ater*, its apical portion is broader, anteriorly expanded, and distinctly convex, with a rounded apical margin; in *P. ghilianii*, it is narrower, nearly flat in frontal view, and more pointed apically (Figure 4A–D). In contrast, females—particularly those with atypical coloration—are more difficult to identify based solely on external morphology.

The pronotal carina remains the most consistent external character distinguishing *P. ater* and *P. ghilianii*, being weakly developed and often restricted to the median region in *P. ater*, but strongly developed and continuous across the pronotum in *P. ghilianii* (Figure 4E,F; [15]). The inclusion of detailed photographs in the present study provides improved visualization of this diagnostic feature.

**Figure 5:**
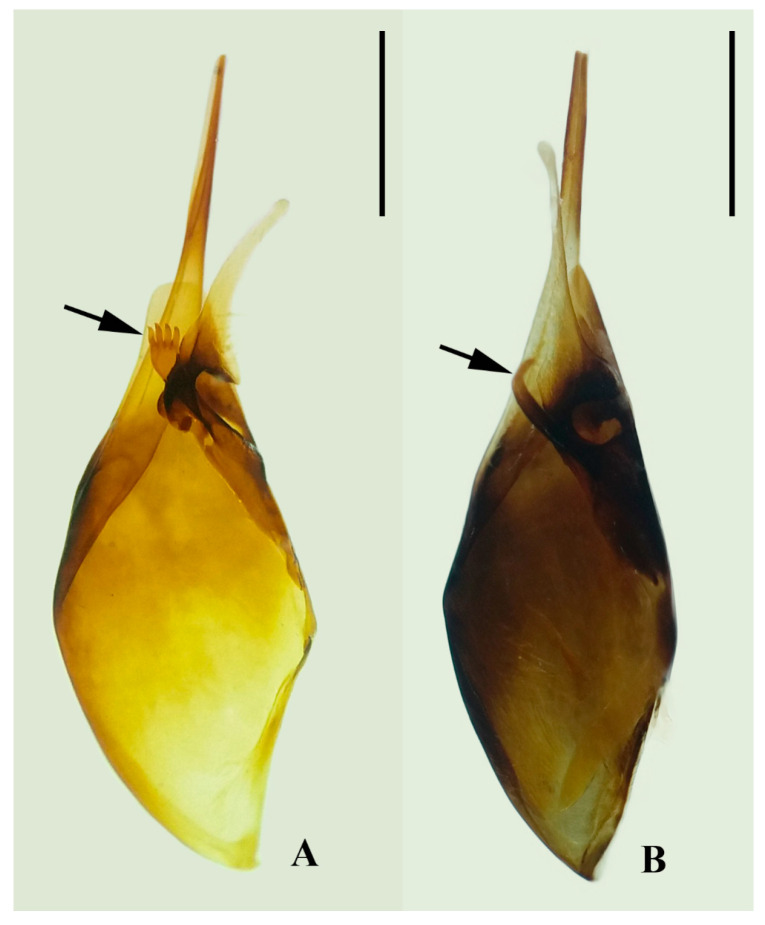
Male genitalia: gonocoxite and volsela. (**A**). *P. ater*; (**B**). *P. ghilianii*. Scale bars: 0.5 mm.

Male genitalia also exhibit informative species-level differences. The apical projection of the gonocoxite clearly separates the species, with *P. ater* showing two to five digitiform projections and *P. ghilianii* only one (Figure 5A,B). This trait, together with the pattern of digitus setation—long, numerous setae in *P. ater* versus short, concentrated setae in *P. ghilianii*—offers robust diagnostic potential.

**Figure 6:**
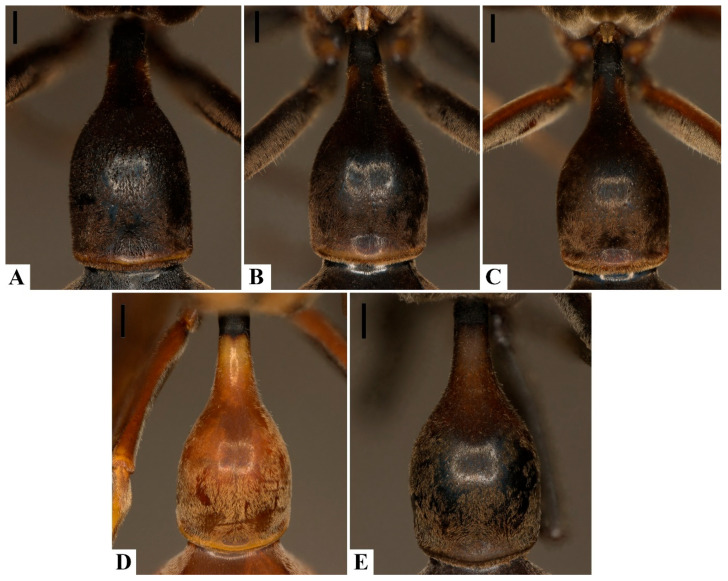
Variation in the presence of punctation on the first metasomal tergum: (**A**–**C**). *Pachymenes ater* de Saussure—(**A**). incipient punctation; (**B**,**C**). punctation present. (**D**,**E**). *Pachymenes ghilianii* (Spinola)—(**D**). punctation absent; (**E**). incipient punctation. Scale bars: 0.5 mm.

Punctation on the first metasomal tergum (T1), although previously proposed as diagnostic, proved unreliable. Both species display overlapping variation in puncture density and depth (Figure 6A–E), and no specimens of *P. ater* exhibited the truly coarse punctation described in earlier works. Such variation appears correlated with general cuticular sculpture and coloration, as melanic specimens tend to show reduced contrast in surface features, occasionally obscuring carinae or sulci.

Overall, our morphological comparison indicates that the shape of the clypeus and male genitalia provide stable diagnostic characters, while the pronotal carina and T1 punctation are more variable and prone to misinterpretation. The figures presented here facilitate accurate identification of *P. ater* and *P. ghilianii* and refine the diagnostic framework for *Pachymenes* species.

### 3.4. Niche Overlap of Pachymenes ater and P. ghilianii

**Figure 7:**
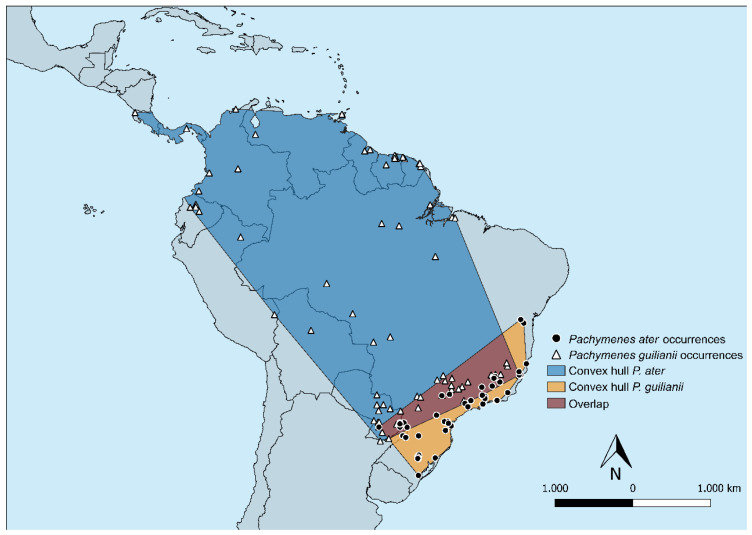
The minimum convex polygon encompassing all occurrences and buffered by 100 km for *Pachymenes ater* and *P. ghilianii*.

**Figure 8:**
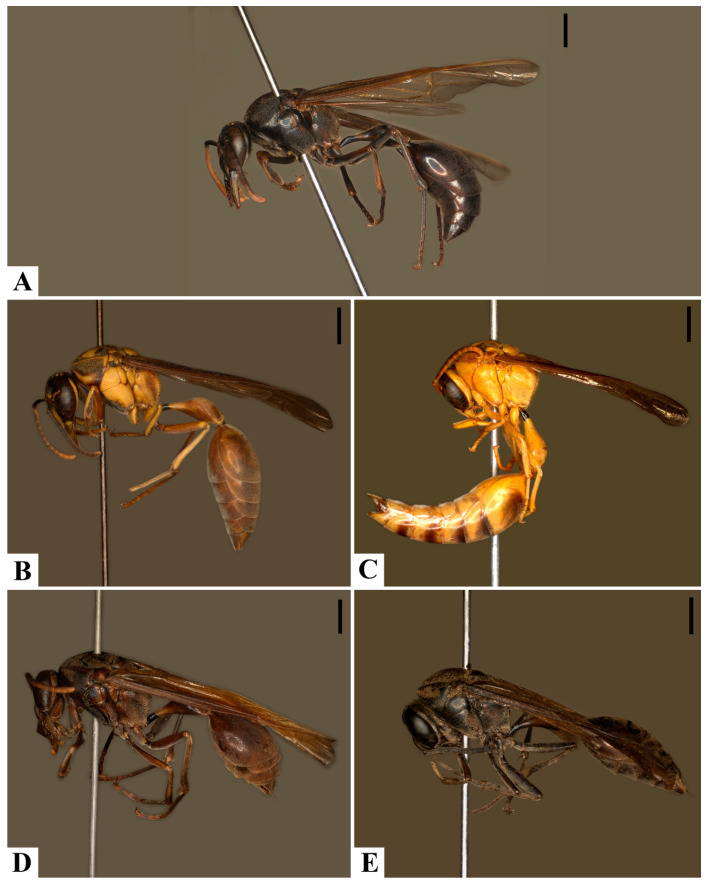
Color variation, habitus: (**A**). *Pachymenes ater* de Saussure; (**B**–**E**). *Pachymenes ghilianii* (Spinola). Scale bars: 2 mm.

Collection records for *P. ater* and *P. ghilianii* are shown in Figure 7, and the chromatic variation can be seen in Figure 8A–E. *Pachymenes ghilianii* exhibits a broader distribution than *P. ater*. Specimens have been recorded from central-southern Brazil, Argentina, and Paraguay, extending northward to the Guianas, Trinidad and Tobago, Ecuador, Colombia, Costa Rica, Panama, and Suriname. In Brazil, the species occurs across multiple biomes, including the Amazon, Atlantic Forest, Cerrado, and Pantanal domains. No records are known from coastal regions of the continent. By contrast, *P. ater* shows a more restricted potential distribution, with collection records concentrated in the southeastern region of South America—specifically from Argentina, Paraguay, and Brazil.

With five variables (BIO01, BIO02, BIO03, BIO12, BIO15), niche overlap between species was moderate, with Schoener’s D = 0.382 (Table 2). The equivalence test did not reject indistinguishable niches (*p* = 0.525), and directional similarity was not significant in either direction (A → B *p* = 0.374; B → A *p* = 0.384) (see Table 2). The PCA explained 70.2% of the variance in PC1 plus PC2 (PC1 = 44.3%; PC2 = 25.9%) (Table 2). In the sensitivity analysis using only BIO01 and BIO12, the metric decreased to D = 0.144, and tests led to the same conclusion (equivalence *p* = 0.602; directional similarities not significant).

There is a partial overlap in the geographical distributions of *P. ater* and *P. ghilianii*. In sympatric populations, however, individuals of each species exhibit distinct body coloration. Specimens of *P. ater* are typically dark brown to blackish, with none or very few pale-yellow markings (Figure 8A). In contrast, individuals identified as *P. ghilianii* generally have brownish integuments with extensive yellow markings (Figure 8B), a pattern observed in most of the examined specimens of this species. Nevertheless, given the wide distribution of *P. ghilianii*, significant intraspecific color variation is observed, ranging from the typical brownish pattern with yellow markings to entirely melanic individuals (Figure 8B–E).

Melanic individuals of *P. ghilianii*—with predominantly dark integument—have been recorded from northern Brazil (Amazonas), Ecuador, Costa Rica, and Peru. In contrast, individuals with the more typical brown-yellow coloration appear to have a broader potential distribution. One male specimen with yellow integument and few brown markings was recorded from Trinidad and Tobago. However, the lack of brown markings in this specimen may be due to its age and preservation state (collected in 1963) (Figure 8C). A single *P. ghilianii* specimen with entirely black integument was observed, originating from Costa Rica (Figure 8E). No other specimens with completely dark integument were examined; all remaining individuals showed at least some brown or yellow markings.

## 4. Discussion

Our primary hypothesis—that *Pachymenes ater* and *P. ghilianii* are reciprocally monophyletic species—was consistently supported across all three phylogenetic methods. All analyses recovered two well-supported monophyletic groups corresponding to each species, using both combined and individual COI and COII datasets. Within *P. ater*, two clades were recovered, but no geographical structure was observed. In contrast, the internal relationships within *P. ghilianii* were less stable and lacked high support, despite broad geographic sampling.

The divergence within *P. ater* is notable given its more restricted distribution and externally uniform morphology. However, previous studies have documented substantial variation in male genitalia in this species [15], supporting our findings. This suggests that genitalic and molecular divergence may occur despite morphological uniformity in external traits. Environmental and biogeographic factors may explain this pattern. As an Atlantic Forest species, *P. ater* may experience genetic isolation due to habitat fragmentation during Pleistocene climate fluctuations [27,52]. A more comprehensive phylogeographic analysis, including targeted sampling and morphometric data, would help test this hypothesis.

Despite ongoing debate about the sole use of COI barcoding for species delimitation [53,54], it remains a useful tool when integrated with other lines of evidence [55]. Our results align with other integrative taxonomic approaches [7,56], particularly under monophyly- and lineage-based species concepts [57,58,59], which regard reciprocal monophyly as a strong criterion for species recognition.

Thus, molecular evidence corroborates the morphological distinction between *P. ater* and *P. ghilianii* proposed in previous taxonomic revisions [14,15]. The mitochondrial markers employed proved effective in distinguishing these species. While we emphasize the importance of integrating morphology, we also advocate for the use of mitochondrial markers in taxonomic studies of Eumeninae.

Although mitochondrial gene trees may not reflect species histories [60], recent analyses based on mitochondrial genome-scale data have recovered well-established topologies within the polistines [61], reinforcing the utility of mitochondrial markers for the understanding of historical relationships. Our results further demonstrate the usefulness of these markers for species delimitation of solitary wasps. DNA extraction from older museum specimens, though challenging due to degradation in dry-pinned insects [62], yielded high-quality sequences for COI and COII.

## 5. Conclusions

Our findings provide robust molecular support for the reciprocal monophyly of *Pachymenes ater* and *P. ghilianii*, confirming their status as distinct species under integrative taxonomic frameworks. Moreover, the detection of divergent lineages within *P. ater*, despite its external morphological uniformity, highlights the importance of incorporating both molecular and morphological data to uncover cryptic diversity. Together, our results reinforce the value of integrative approaches in clarifying species limits in morphologically conserved insect groups.

## Figures and Tables

**Table 1:** Morphological differences between *Pachymenes ghilianii* (Spinola) and *Pachymenes ater* de Saussure. Characters that allow unequivocal identification of the two species are highlighted with an asterisk.

Morphological Features	Character States	
	*Pachymenes ater*	*Pachymenes ghilianii*
Pronotal carina *	Weakly developed	Present along the entire dorsal surface
Sculpture of T1	Coarse punctuation apically at middle	Smooth, with inconspicuous punctures
Outer margin of male clypeus, lateral view *	Truncate	Acutely angled
Projection on gonocoxite *	Two to five apical projections	With one simple apical projection
Setae on digitus *	Long	Short

**Table 2:** Niche overlap and hypothesis tests in PCA-env for two predictor setups: A, five variables (BIO01, BIO02, BIO03, BIO12 and BIO15) and B, two variables (BIO01 and BIO12). The table reports Schoener’s D, *p*-values for the niche equivalence test, directional niche similarity (A?B and B?A), and the proportion of variance explained by PC1, PC2 and PC1 + PC2. Non-significant *p*-values indicate no evidence against the corresponding null hypotheses.

Setup	Schoener’s D	Equivalence *p*	Similarity *p* (A-B)	Similarity (B-A)	PC1 Percent	PC2 Percent	PC1 + PC2 Percent
A	0.382033	0.52495	0.374052	0.383832	44.341354	25.856061	70.197415
B	0.144146	0.601597	0.598403	0.608184	70.589515	29.410485	100

## Data Availability

The original contributions presented in this study are included in the article and in the Supplementary Material (SM) file. Genbank accession numbers are also presented in the SM. Further inquiries can be directed to the corresponding author.

## Supplementary Materials

The following supporting information can be downloaded at: https://www.mdpi.com/article/10.3390/insects17010078/s1, Figure S1: Strict consensus of four equally parsimonious cladograms (length = 139; consistency index = 0.89; retention index = 0.96) based on equal character weighting of the COI sequences. Support values were estimated using bootstrap (1000 replicates). Black circles indicate synapomorphies; white circles indicate homoplasies; Figure S2: Strict consensus of four equally parsimonious cladograms (length = 166; consistency index = 0.92; retention index = 0.96) based on equal character weighting of the COII sequences. Support values were estimated using bootstrap (1000 replicates). Black circles indicate synapomorphies; white circles indicate homoplasies; Figure S3: Maximum likelihood phylogeny based on 472 bp of the mitochondrial gene COI. Numbers on branches indicate bootstrap support (1000 replicates); Figure S4: Maximum likelihood phylogeny based on 617 bp of the mitochondrial gene COII. Numbers on branches indicate bootstrap support (1000 replicates); Figure S5: Bayesian inference phylogeny based on 472 bp of the mitochondrial gene COI. Numbers on branches indicate posterior probability values; Figure S6: Bayesian inference phylogeny based on 617 bp of the mitochondrial gene COII. Numbers on branches indicate posterior probability values; Table S1: List of specimens used in the molecular analyses, with their respective code, identification, year of collection, locality and geographic coordinates; Table S2: Primer specification and PCR conditions used in the amplification of the target genes; Table S3: List of samples (specimens) and amplification success for different mitochondrial gene sequences: Cytochrome Oxidase Subunit I (COI); Cytochrome oxidase Subunit II (COII). Samples for which amplification was successful are referenced with their respective Genbank accession number; Table S4: Substitution models selected for each codon position of the two sequenced mitochondrial loci.
