# Supplementary material for: Integrative Taxonomy Clarifies Species Limits in Two Closely Related Solitary Wasps: Pachymenes ater and Pachymenes ghilianii (Hymenoptera: Vespidae: Eumeninae)
Source: Insects. 2026 Jan 9;17(1):78. doi: 10.3390/insects17010078 (PMC12842564; doi:10.3390/insects17010078)
Supplement: Supplementary file 1 [file insects-17-00078-s001.zip › insects-4070012-supplementary.pdf]

Supplementary Material

For

Integrative taxonomy clarifies species limits in two closely related solitary wasps: *Pachymenes ater* and *Pachymenes ghilianii* (Hymenoptera, Vespidae, Eumeninae)

Wellington D. Ferreira[1, 2, *], Rodolpho S. T. Menezes[3], Matheus C. Viana[3], Marcel G. Hermes[1]

[1]Centro de Estudos em Sistemática e Biologia de Insetos, Centro de Biodiversidade e Patrimônio Genético, Instituto de Ciências Naturais, Universidade Federal de Lavras, Lavras, Brazil; marcelhermes@ufla.br (MGH)

[2]Instituto Federal de Educação, Ciência e Tecnologia Baiano – Campus Guanambi, Zona Rural, Distrito de Ceraíma, Bahia, Brazil; wellington.ferreira@ifbaiano.edu.br (WDF)

[3]Departamento de Ciências Biológicas, Universidade Estadual de Santa Cruz, Ilhéus, Brazil; rstmenezes@gmail.com (RSTM); matheusmakio120@gmail.com (MCV)

[*]Correspondence: wellington.ferreira@ifbaiano.edu.br (WDF)

Table S1. List of specimens used in the molecular analyses, with their respective code, identification, year of collection, locality and geographic coordinates.

Code	Taxon	Year of Collection	Locality	Geographic Coordinates
PAAT01	*P. ater*	2016	Brasil, MG, Ingaí	21°20'47"S 44°59'27"O
PAAT02	*P. ater*	2016	Brasil, MG, Ingaí	21°20'47"S 44°59'27"O
PAAT03	*P. ater*	2011	Brasil, SP, Ribeirão Grande	24°16'28"S 48°25'20"O
PAAT04	*P. ater*	2015	Brasil, MG, Ingaí	21°20'47"S 44°59'27"O
PAAT05	*P. ater*	2016	Brasil, MG, Ingaí	21°20'47"S 44°59'27"O
PAAT06	*P. ater*	2008	Brasil, PR, Curitiba	25°25'47"S 49°16'19"O
PAAT07	*P. ater*	2015	Brasil, MG, Ingaí	21°20'47"S 44°59'27"O
PAGH01	*P. ghilianii*	2013	Brasil, MG, Itanhandu	22°17'45"S 44°56'06"O
PAGH02	*P. ghilianii*	2007	Paraguai, Concepcion	23°24'13"S 56°31'18"O
PAGH03	*P. ghilianii*	2008	Brasil, AM, Presidente Figueiredo	02°03'44"S 59°58'28"O
PAGH04	*P. ghilianii*	2012	Brasil, RO, Itapuã do Oeste	09°12'08"S 63°10'48"O
PAGH05	*P. ghilianii*	2011	Brasil, AM, Iranduba	03°17'06"S 60°11'09"O
PAGH06	*P. ghilianii*	2011	Brasil, AM, Iranduba	03°17'06"S 60°11'09"O
PAGH07	*P. ghilianii*	2015	Brasil, MG, Ingaí	21°20'47"S 44°59'27"O
PAGH08	*P. ghilianii*	2012	Brasil, RO, Itapuã do Oeste	09°12'08"S 63°10'48"O
PAGH09	*P. ghilianii*	2009	Brasil, SP, Barretos	20°33'25"S 48°34'04"O
PAGH10	*P. ghilianii*	2015	Brasil, MG, Ingaí	21°20'47"S 44°59'27"O
PAGH11	*P. ghilianii*	1985	Peru, Madre de Dios	12°37'09"S 70°23'37"O
PAGH12	*P. ghilianii*	1976	Equador, Napo	0°59'20"N 77°48'57"O
PAGH13	*P. ghilianii*	2008	Brasil, SP, Matão	21°36'10"S 48°21'57"O
PAGH14	*P. ghilianii*	2015	Brasil, MG, Ingaí	21°20'47"S 44°59'27"O
PASE01	*P. sericeus*	2009	Brasil, MS, Bonito	21°03'28"S 56°43'52"O

Table S2. Primer specification and PCR conditions used in the amplification of the target genes.

Primers		PCR Conditions
**COI**		95°C; 30s; 45°C; 1min; 72°C; 1min - 35 cycles
CI-J-1718	GGAGGATTTGGAAATTGATTAGTTCC	
CI-N-2191	GGTAAAATTAAAATATAAACTTC	
**COII**		94°C; 30s; 50°C; 45s; 72°C; 45s - 40 cycles
E2	GGCAGAATAAGTGCATTG	
COII1-2	ATTTTATACCACAAATTTCTGAACATTG	

Table S3. List of samples (specimens) and amplification success for different mitochondrial gene sequences: Cytochrome Oxidase Subunit I (COI); Cytochrome oxidase Subunit II (COII). Samples for which amplification was successful are referenced with their respective Genbank accession number.

Sample Details			Molecular Marker	
**Code**	**Species**	**Storage**	**COI**	**COII**
PAAT01	*P. ater*	Alcohol (90%)	PX649452	PX685881
PAAT02	*P. ater*	Alcohol (90%)	-	PX685884
PAAT03	*P. ater*	Dry-Mounted	PX649449	PX685878
PAAT04	*P. ater*	Dry-Mounted	PX649453	PX685882
PAAT05	*P. ater*	Dry-Mounted	PX649454	PX685883
PAAT06	*P. ater*	Dry-Mounted	PX649450	PX685879
PAAT07	*P. ater*	Dry-Mounted	PX649451	PX685880
PAGH01	*P. ghilianii*	Dry-Mounted	PX649444	PX685875
PAGH02	*P. ghilianii*	Dry-Mounted	PX649447	PX685876
PAGH03	*P. ghilianii*	Dry-Mounted	PX649445	-
PAGH04	*P. ghilianii*	Dry-Mounted	PX649442	-
PAGH05	*P. ghilianii*	Dry-Mounted	PX649446	-
PAGH06	*P. ghilianii*	Dry-Mounted	PX649441	-
PAGH07	*P. ghilianii*	Dry-Mounted	PX649440	PX685874
PAGH08	*P. ghilianii*	Dry-Mounted	PX649443	-
PAGH09	*P. ghilianii*	Dry-Mounted	PX649439	PX685873
PAGH10	*P. ghilianii*	Dry-Mounted	-	-
PAGH11	*P. ghilianii*	Dry-Mounted	-	-
PAGH12	*P. ghilianii*	Dry-Mounted	-	-
PAGH13	*P. ghilianii*	Dry-Mounted	-	-
PAGH14	*P. ghilianii*	Dry-Mounted	-	-
PASE01	*P. sericeus*	Alcohol (90%)	PX649448	PX685877

Table S4. Substitution models selected for each codon position of the two sequenced mitochondrial loci.

<table>
<tr><th></th><th colspan="3">Codon Position</th></tr>
<tr><th>Locus</th><th>1</th><th>2</th><th>3</th></tr>
<tr><td>COI</td><td rowspan="2">HKY85</td><td rowspan="2">F81</td><td rowspan="2">GTR + I</td></tr>
<tr><td>COII</td></tr>
</table>

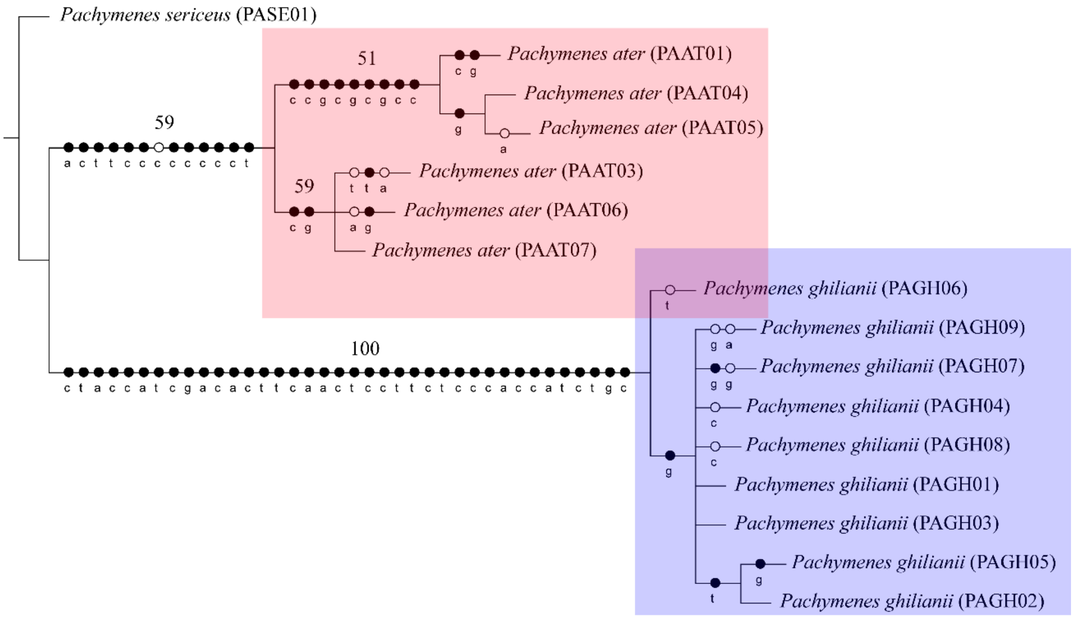

Figure S1. Strict consensus of four equally parsimonious cladograms (length = 139; consistency index = 0.89; retention index = 0.96) based on equal character weighting of the COI sequences. Support values were estimated using bootstrap (1000 replicates). Black circles indicate synapomorphies; white circles indicate homoplasies.

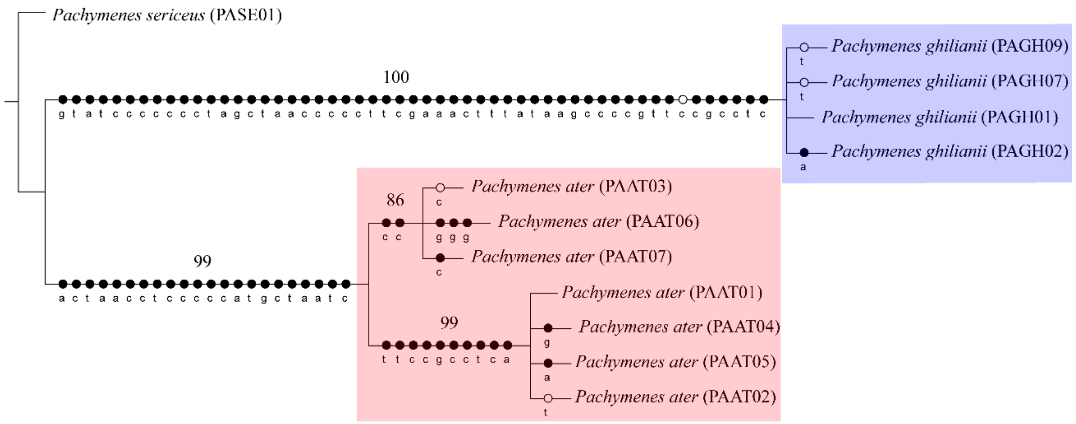

Figure S2. Strict consensus of four equally parsimonious cladograms (length = 166; consistency index = 0.92; retention index = 0.96) based on equal character weighting of the COII sequences. Support values were estimated using bootstrap (1000 replicates). Black circles indicate synapomorphies; white circles indicate homoplasies.

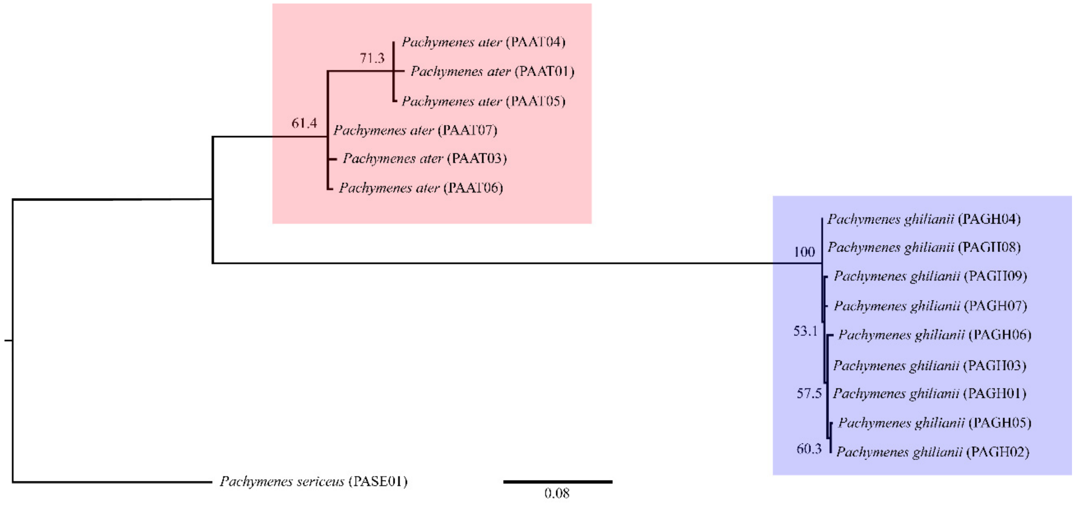

Figure S3. Maximum likelihood phylogeny based on 472 bp of the mitochondrial gene COI. Numbers on branches indicate bootstrap support (1000 replicates).

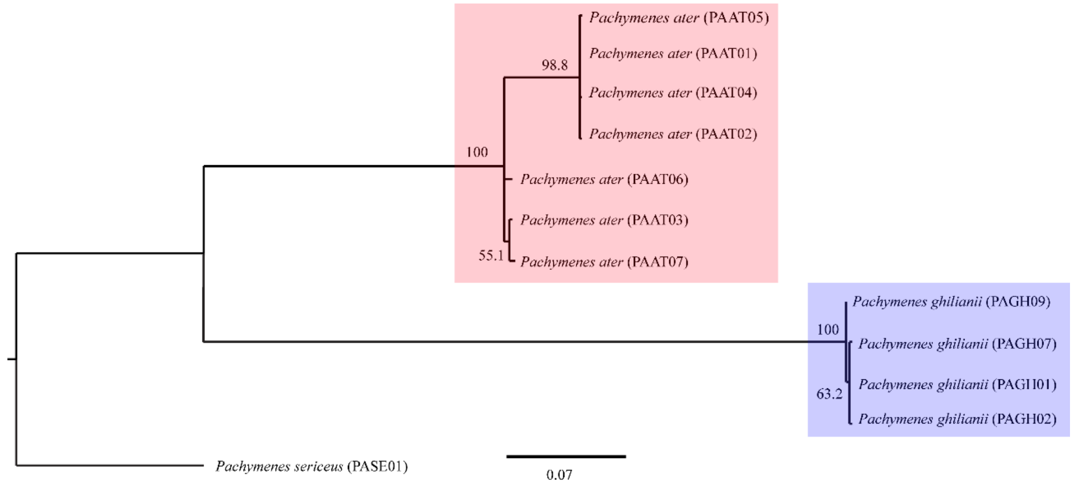

Figure S4. Maximum likelihood phylogeny based on 617 bp of the mitochondrial gene COII. Numbers on branches indicate bootstrap support (1000 replicates).

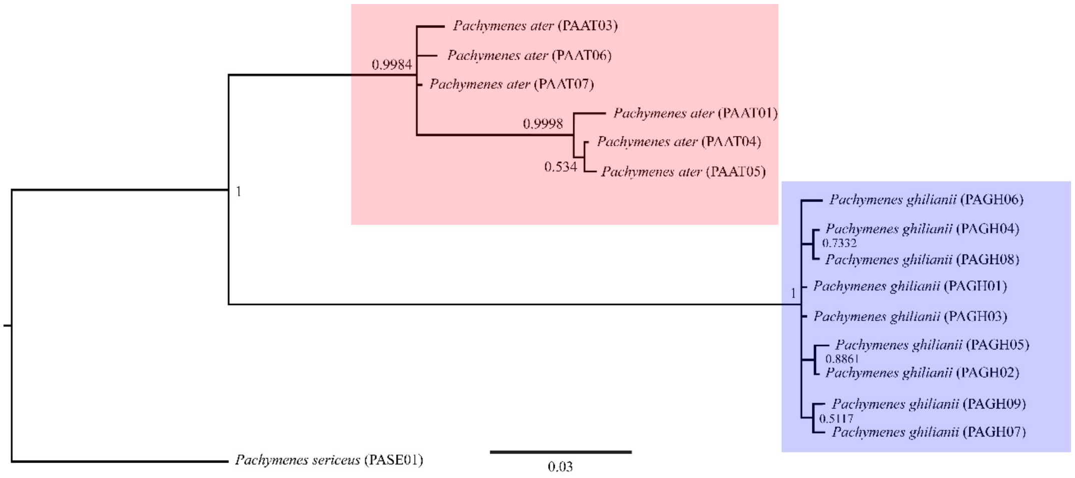

Figure S5. Bayesian inference phylogeny based on 472 bp of the mitochondrial gene COI. Numbers on branches indicate posterior probability values.

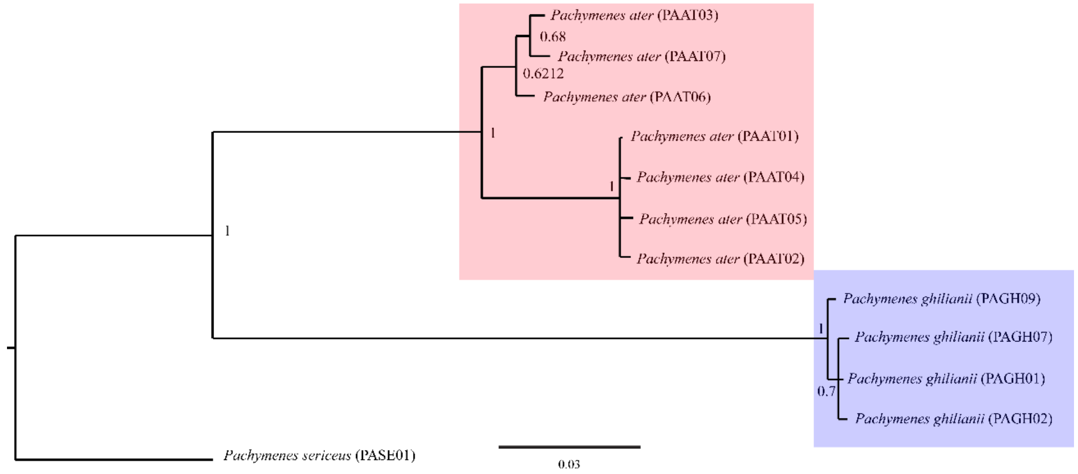

Figure S6. Bayesian inference phylogeny based on 617 bp of the mitochondrial gene COII. Numbers on branches indicate posterior probability values.

Specimens Examined in the Morphological Studies

The information provided in quotation marks in this section is a transcription of the labels associated with the specimens. Quotation marks indicate different labels for the same specimen, and backslashes (\) indicate different lines on the same label.

Pachymenes ater de Saussure

♀♀

1 ♀, CEUFLA: “Brasil, Paraná, Quatro\ Barras, Trilha Anhagava\ 25°23’S 49°00’W\15.x.2007, KS, Ramos *leg*” “Pachymenes\ ater Sss. ♀\ Grandinete, Y. C. det 2014” (partially handwriten label); 1 ♀, CEUFLA: “Brasil, Paraná\ Piraquara\ ii. 2006\ P. C. Grossi col.” “Pachymenes\ ater Sss. ♀\ Grandinete, Y. C. det 2014” (partially handwriten label); 1 ♀, CEUFLA: “BRASIL, Paraná, Tibagi\ Parque Estadual Guartelá,\ 09-11-XI-2007, 900 m,\ Manuel, P.C. Grossi col.” “Pachymenes\ ater Sss. ♀\ Grandinete, Y. C. det 2014” (partially handwriten label); 1 ♀, CEUFLA: “Brasil, Paraná, Piraquara\ Mananciais da Serra\ 30.X.2006 P. Grossi col.” “Pachymenes\ ater Sss. ♀\ Grandinete, Y. C. det 2014” (partially handwriten label); 1 ♀, CEUFLA: “BRASIL, Paraná, Piraquara,\ Manciais da Serra\ 22.x.2008, 25°29’S\ 48°58’W P.C. Grossi (leg.)” “Pachymenes\ ater Sss. ♀\ Grandinete, Y. C. det 2014” (partially handwriten label); 1 ♀, CEUFLA: “Brasil RJ 6 km a NW\ de Itatiaia Pq Nac\ Itatiaia Hotel Simon 1100 m\ 22436°S 44609°W\ 28.x.2011 M. G. Hermes” “Pachymenes\ ater Sss. ♀\ Grandinete, Y. C. det 2014” (partially handwriten label); 1 ♀, CEUFLA: “Brasil, Minas

Gerai\ Passa Quatro, Flona\ 22°23′S 44°56′W\ 09.iii.2013\ M.G. Hermes col." "Pachymenes\ ater Sss. ♀\ Grandinete, Y. C. det 2014" (partially handwriten label); 1 ♀, CEUFLA: "Brasil, Paraná\ Piraquara\ ii.2006\ P. C. Grossi col." "Pachymenes ♀\ ater Sss.\ Grandinete, Y. C. det. 2014" (partially handwriten label); 1 ♀, CEUFLA: "Brasil, MG/Ingaí,\ Res. Biol. Boqueirão\ Mata ciliar 21°20′47″S\ 44°59′27″W, 27.x.2009\ M.H. Simões col."; 1 ♀, CEUFLA: "Brasil, MG/Ingaí,\ Res. Biol. Boqueirão\ Mata ciliar 21°20′47″\ 44°59′27″W, 18.viii.2009\ M.H. Simões col."; 1 ♀, CEUFLA: "Brasil RJ 6km a NW\ de Itatiaia Pq Nac\ Itatiaia Hotel Simon 1100m\ 22 436°S 44609°W. 28.x.2011 M. G. Hermes" "Pachymenes ♀\ ater SSs\ Grandinete, Y. C. det. 2014" (partially handwriten label); 1 ♀, CEUFLA: "Brasil, PR, Quatro\ Barras, Morro\ Anhangava 11.III.2010\ M. G. Hermes col." "Pachymenes ♀\ ater SSs\ Grandinete, Y. C. det. 2014" (partially handwriten label); 1 ♀, CEUFLA: "Brasil, Paraná\ Piraquara\ 22.xi.2005\ M. G. Hermes col." "Pachymenes ♀\ ater SSs\ Grandinete, Y. C. det. 2014" (partially handwriten label); 1 ♀, CEUFLA: "Brasil, MG/Ingaí\ Res. Biol. Boqueirão\ Mata ciliar 21°20′47″S\ 44°59′27″W. 27.x.2009\ M. H. Simões col."; 1 ♀, CEUFLA: "Brasil, MG/Ingaí\ Res. Biol. Boqueirão\ Mata ciliar 21°20′47″S\ 44°59′27″W. 27.x.2009\ M. H. Simões col."; 1 ♀, CEUFLA: "Brasil, Minas Gerais\ Itanhandu\ 22°17′S 44°56′W\ 10.iii.2013\ M. G. Hermes col." "Pachymenes ♀\ ater SSs\ Grandinete, Y. C. det. 2014" (partially handwriten label); 1 ♀, CEUFLA: "Brasil, Paraná, Piraquara\ Manciais da Serra\ 30.X.2006 P. Grossi col." "Pachymenes ♀\ ater SSs\ Grandinete, Y. C. det. 2014" (partially handwriten label); 1 ♀, CEUFLA: "Brasil, Minas Gerais\ Passa Quatro, Flona\ 22°23′S 44°56W\ 09.iii.2013\ M. G. Hermes col." "Pachymenes ♀\ ater SSs\ Grandinete, Y. C. det. 2014" (partially handwriten label); 1 ♀, DZSJRP: "Brasil, SP, Jundiaí\ Serra do Japi\ 14.x.2008\ Antiqueira, P. A. P. col." "EUM0367"; 1 ♀, DZSJRP: "Brasil, MG/Ingaí\ Res. Biol. Boqueirão\ Mata ciliar 21°20′47″S\ 44°59′27″W 01.v.2009\ M. H. Simões col."; 1 ♀, DZSJRP: "BRASIL, Paraná, Piraquara\ Mananciais da Serra\ 08.xi.2008, 25°29′S\ 48°58′W P. C. Grossi (leg.)" "Pachymenes ♀\ ater SSs\ Grandinete, Y. C. det. 2014" (partially handwriten label); 1 ♀, DZSJRP: "Brazil, PR, Curitiba\ Rio Passaúna\ 29.III.2008\ G.A.R. Melo col." "Ninho Paraná" (handwriten label); 1 ♀, CEUFLA: "Brasil, Minas Gerais\ Ingaí, Res. Biol.\ Boqueirão 21° 20′47′′S\ 44° 59′ 27′′ W, 08.x.2015\ E. P. Pires col."; 1 ♀, DZSJRP: "Brasil, SP, Jundiaí\ Serra do Japi\ 14.x.2008\ Antiqueira, P.A.P. col." "EUM0369"; 1 ♀, AMNH: "Nova Teutonia\ Santa Cararina\ BRAZIL II-65\ Fritz Plaumann" (partially handwriten label) "Pachymenes ♀\ ater sss.\ Y. C. Grandinete det. 2015" (partially handwriten label); 1 ♀, AMNH: "ARG. -MISIONES\ Dos de Mayo\ Fritz – XII. 73" "*Pachymenes ater*\ de Saussure, 1852\ Y. C. Grandinete det. 2014"; 1 ♀, CEUFLA: "Brasil, Minas Gerais\ Ingaí, Res. Biol.\ Boqueirão 21° 20′47′′S\ 44° 59′ 27′′ W, 08.x.2015\ E. P. Pires col."; 1 ♀, DZSJRP: "Brasil: SP, Gália e\ Alvinlândia\ Est. Ecol. Caetetus\ 24. ii. 2005\ Coleta ativa"

"EUM0366" (in the back of the label); 1 ♀, DZSJRP: "Brasil, Minas Gerais\ Ingaí, Res. Biol.\ Boqueirão 21° 20'47''S\ 44° 59' 27'' W, 08.x.2015\ E. P. Pires col."; 1 ♀, DZSJRP: "Brazil, PR, Curitiba\ Rio Passaúna\ 29.III.2008\ G.A.R. Melo col." "Ninho Paraná" (handwriten label); 1 ♀, CEUFLA: "Brasil, Paraná\ Piraquara\ ii.2006\ P. C. Grossi col." "Pachymenes ♀\ ater Sss.\ Grandinete, Y. C. det. 2014"; 2 ♀♀, CESC/UNISC: "BRA-RS-Santa Cruz do Sul\ Cinturão Verde 01.04.2004\ leg: Marcel G. Hermes" "Pachymenes\ ater Sauss., 1852\ M. G. Hermes det., 2004"; 2 ♀♀, CESC/UNISC: "BRA-RS-Santa Cruz do Sul\ Cinturão Verde 05.03.2004\ leg: Marcel G. Hermes" "Pachymenes\ ater Sauss., 1852\ M. G. Hermes det., 2004"; 2 ♀♀, CESC/UNISC: "BRA-RS-Santa Cruz do Sul\ Cinturão Verde 29.iv.2004\ leg: Marcel G. Hermes" "Pachymenes\ ater Sauss., 1852\ M. G. Hermes det., 2004"; 1 ♀, CESC/UNISC: "BRA-RS-Santa Cruz do Sul\ Cinturão Verde 02.03.2004\ leg: Marcel G. Hermes" "Pachymenes\ ater Sauss., 1852\ M. G. Hermes det., 2004"; 1 ♀, CESC/UNISC: "BRA-RS-Santa Cruz do Sul\ Cinturão Verde 06.III.2004\ leg: Dione J. Krise" "Pachymenes\ ater Sauss., 1852\ M. G. Hermes det., 2004"; 1 ♀, CESC/UNISC: "BRA-RS-Santa Cruz do Sul\ Cinturão Verde 05.02.2002\ leg: Dione J. Krise" "Pachymenes\ ater Sauss., 1852\ M. G. Hermes det., 2004" (partially handwriten label)

♂♂

3 ♂♂, DZSJRP: "Brazil, PR, Curitiba\Rio Passaúna\ 29.III.2008\ G. A. R. Melo col." "Ninho Paraná" (handwriten label) (genitalia extracted); 1 ♂, AMNH: "BRAZIL: SP\ Est. Biol. Boracéia\ 23°39'S, 45°53'W\ 909 m, 2-5 May 1997\ James M. Carpenter" "Pachymenes\ ater\ Saussure ♂\ det. J. M. Carpenter" (partially handwriten label) (genitalia extracted); 2 ♂♂, CEUFLA: "BRASIL, Paranáz Curitiba, Rio Passaúna\ 29.iii.2008,\ G. A. R. Melo (leg.)" "Pachymenes ♂\ ater Sss.\ Grandinete, Y. C. det. 2014" (partially handwriten label); 1 ♂, CEUFLA: "Brasil, Paraná, Piraquara\ Mananciais da Serra\ 12.II.2007 P. Grossi col." "Pachymenes ♂\ ater Sss.\ Grandinete, Y. C. det. 2014" (partially handwriten label); 2 ♂♂, LSBI/ULFA: "Brasil, SP, 5km a SWz de S. J. Barreiro, Serra\ da Bocaina 1050m\ 22.675°S 44.613°W\ 22.x.2011 M. G. Hermes" "Pachymenes ♂\ ater Sss.\ Grandinete, Y. C. det. 2014" (partially handwriten label) (genitalia extracted); 1 ♂, CEUFLA: "Brasil, Minas Gerais\ Passa Quatro, Flona\ 22°23'S 44°56'W\ 09.iii.2013\ M. G. Hermes col." "Pachymenes ♂\ ater Sss.\ Grandinete, Y. C. det. 2014" (partially handwriten label) (genitalia extracted); 1 ♂, CEUFLA: "Brazil, PR, Tibagi\ P. E. Guartelá\ III.2010 Grossi &\ Parizotto cols." "Pachymenes ♂\ ater Sss.\ Grandinete, Y. C. det. 2014" (partially handwriten label); 1 ♂, CESC/UNISC: "BRA-RS-Santa Cruz do Sul\ Cinturão Verde 05.02.2002\ leg: Dione J. Krise" (partially handwriten label) "N: 2364 L: 02\ P: 31 H: 15:00" (partially

handwriten label) “Pachymenes\ ater Sauss., 1852\ M. G. Hermes det., 2004” (genitalia extracted).

***Pachymenes ghilianii* (Spinola)**

♀♀

1 ♀, DZSJRP: “Brasil, SP, Paulo de\ Faria\ 30.iii.2006\ Gomes, B. col.” “EUM0323”; 1 ♀, DZSJRP: “Brasil, SP, Paulo de\ Faria\ 30.iii.2006\ Gomes, B. col.” “EUM0321”; 1 ♀, DZSJRP: “Brasil, SP, Paulo de\ Faria\ 30.iii.2006\ Gomes, B. col.” “EUM0320”; 1 ♀, DZSJRP: “Brasil, SP, Matão\ 22.x.2008\ Coleta ativa\ Tanaka, G. M. col.” “EUM0337”; 1 ♀, DZSJRP: “Brasil, SP, Pindorama\ 23.ii.2011\ Coleta ativa\ Capusso, O. L. col.” “EUM0336”; 1 ♀, DZSJRP: “Brasil, SP, Matão\ 09.i.2008\ Coleta ativa\ Tanaka, G. M. col.” “EUM0333”; 1 ♀, DZSJRP: “Brasil, SP, Pindorama\ 02.ii.2010\ Coleta ativa\ Capusso, O. L. col.” “EUM0332”; 1 ♀, DZSJRP: “Brasil, SP, Paulo de\ Faria\ 02.ii.2006\ Gomes, B. col.” “EUM0325”; 1 ♀, DZSJRP: “Brasil, SP, Matão\ 28.vi.2007\ Noll, F. B. col” “EUM0308”; 1 ♀, DZSJRP: “Brasil, SP, Matão\ 01.ii.2007\ Noll, F. B. col” “EUM0309”; 1 ♀, DZSJRP: “Brasil, SP, Paulo de\ Faria\ 30.iii.2006\ Gomes, B. col.” “EUM0312”; 1 ♀, DZSJRP: “Brasil, SP, Paulo de\ Faria\ 18.v.2006\ Gomes, B. col.” “EUM0317”; 1 ♀, DZSJRP: “Brasil, SP, Paulo de\ Faria\ 27.iv.2006\ Gomes, B. col.” “EUM0318”; ♀, DZSJRP: “Brasil, SP, Paulo de\ Faria\ 22.iii.2005\ Gomes, B. col.” “EUM0319”; 1 ♀, DZSJRP: “Brasil, SP, Paulo de\ Faria\ 30.iii.2006\ Gomes, B. col.” “EUM0326”; 1 ♀, DZSJRP: “Brasil, SP, Paulo de\ Faria\ 11.v.2006\ Gomes, B. col.” “EUM0327”; 1 ♀, DZSJRP: “Brazil: RO, Itapuã do Oeste\ Floresta Nacional do Jamari\ 02.ix.2012\ Coleta ativa\ Gomes, B. col.” “EUM0328”; 1 ♀, DZSJRP: “Brazil: SP, Luiz Antônio\ Est. Ecol. Jataí (557m)\ 21°33’42”W/47°48’37”S\ 08.xi.2011 – Coleta ativa\ Grandinete, Y. C. col.” “EUM0345”; ♀, DZSJRP: “Brazil: RO, Porto Velho\ Universidade Federal de\ Rondônia\ 05.viii.20112 – Coleta Ativa\ Gomes, B. col.” “EUM0341”; 1 ♀, AMNH: “PERU: Madre de Dios\ Tambopata Wild. Res.\ 12°51’S,69 17’W, 290m\ 22-25 January, 1985\ J. Carpenter & D. Bowers” “*Pachymenes ghilianii*\ (Spinola, 1851)\ Y. C. Grandinete det. 2014”; 1 ♀, DZSJRP: “Brasil, SP, Matão\ 15.iv.2008\ Noll, F. B. col” “EUM0310”; 1 ♀, FFCLRP/USP: “F: Eumenidae\ S.F.:” (partially handwriten label) “Loc: Cajuru SP\ Dat: 23-VI-984\ Col: Lacerda L. M.” (handwriten label); 1 ♀, AMNH: “PARAGUAY: San\ Pedro. Rio Ypane\ Cororo\ XII.1983\ M. A. Fritz” “*Pachymenes ghilianii*\ (Spinola, 1851)\ Y. C. Grandinete det. 2014”; 1 ♀, AMNH: “PARAGUAY: San\ Pedro – Rio Ypane\ Cororo\ XII.1983\ M. A. Fritz” “*Pachymenes ghilianii*\ (Spinola, 1851)\ Y. C. Grandinete det. 2014”; 1 ♀, AMNH: “Bartica, BG\ V.13.1901” “Pachyemnes\ ghilianii\ (Spinola)\ Det. J. M. Carpenter”; 1 ♀, AMNH: “BRITSH GUIANA\ Karabo, Bartica\ Dist. 1921”

“Trop. Research Station\ New York Zool. Society\ No. 21/07” (partially handwriten label) “Gift of New York\ Zoo. Soc. Dept.\ Tropical Research\ William Beebe, Dir.” “Pachymenes\ ghilianii (Spinola)\ ♀ pallipes Sauss.\ det. J. v. Vecht 1976” “Pachymenes ♀\ ghilianii (Spin.)\ Y. C. Grandinete det. 2015” (partially handwriten label); 1 ♀, DZSJRP: “Brasil, SP, Paulo de\ Faria\ 13.iv.2006\ Gomes, B. col.” “EUM0314”; 1 ♀, AMNH: “San Jose\ (Costa Rica)” “425” “Pachymenes ♀\ ghilianii (Spin.)\ Y. C. Grandinete det. 2015” (partially handwriten label); 1 ♀, FFCLRP/USP: “Parque Est. Morro do Diabo\ Teodoro Sampaio-SP, Brasil\ 22°32’33”S, 52°19’40”W\ 14-16/11/1999 – Tavares leg.”; 1 ♀, AMNH: “Belem, Para\ Brazil\ 5-V-1967\ Coll. Y. Sedman” “Pachymenes ♀\ ghilianii (Spin.)\ Y. C. Grandinete det. 2015” (partially handwriten label); 1 ♀, DZSJRP: “Brazil: SP, Onda Verde\ Faz. Fischer\ 07.vi.2010\ Coleta ativa\ Capusso, O.L. col.” “EUM0335”; 1 ♀, DZSJRP: “Brazil: SP, Onda Verde\ Faz. Fischer\ 17.xii.2009\ Coleta ativa\ Capusso, O.L. col.” “EUM0334”; 1 ♀, DZSJRP: “Brazil: SP, Gália e\ Alvinlândia\ Est. Ecol. Caetetus\ 24.ii.2005\ Coleta ativa” “EUM0340”; 1 ♀, DZSJRP: “Brasil, SP, Paulo de\ Faria\ 13.iv.2006\ Gomes, B. col.” “EUM0324”.

♂♂

1 ♂, DZSJRP: “Brazil: SP, Pindorama\ 06.viii.2009\ Coleta ativa\ Capusso, O.L. col.” “EUM0331”; 1 ♂, DZSJRP: “Brazil, RO, Itapuã do Oeste\ Floresta Nacional do Jamari\ 18.viii.2012\ Coleta ativa\ Gomes, B. col.” (genitalia extracted); 1 ♂, AMNH: “PARAGUAY: San\ Pedro, Rio Ypane\ Cororo\ XII.1983 (partially handwriten label) “*Pachymenes ghilianii*\ (Spinola, 1851)\ Y. C. Grandinete det. 2014”; 1 ♂, CEUFLA: “BRASIL, AM, Presidente\ Figueiredo, AM 240 km 11\ Pousada Berro D’Água\ 23.viii.2008, 135 m, 2°03’44”S\ 59°58’28”W M. G. Hermes” “Pachymenes ♂\ ghilianii (Spin.)\ Grandinete, Y. C. det. 2014”; 1 ♂, DZSJRP: “Brazil: SP, Matão\ 22.x.2008\ Coleta ativa\ Tanaka, G. M. col.” “EUM0338”; 1 ♂, AMNH, “Restrepo\ Dept. Meta\ 500 M.” “Colombia\ 1936” “J. Bequaert\ Collector” “*Pachymenes ghilianii*\ (Spinola)\ Y. C. Grandinete det. 2014” (genitalia extracted); 1 ♂, AMNH, “3” “BRAZIL: Est. Minas Gerais:\ Dionísio, 4 yr-old eucalyptus\ platation, 21 April 2008\ R. Silva-Filho” (genitalia extracted); 1 ♂, DZSJRP: “Brazil: SP, Pindorama\ 02.ii.2010\ Coleta ativa\ Capusso, O. L. col.” “EUM0343” (genitalia extracted); 1 ♂, DZSJRP: “Brasil, SP, Paulo de\ Faria\ 13.iv.2006\ Gomes, B. col.” “EUM0311” (genitalia extracted); 1 ♂, DZSJRP: “Brazil: SP, Magda\ 15.vii.2008\ Coleta ativa\ Tanaka, G. M. col.” “EUM0342” (genitalia extracted).
